# The role of metals in cancer and the use of metal chelators as a potential treatment strategy

**DOI:** 10.1007/s10534-026-00847-1

**Published:** 2026-07-20

**Authors:** M. Fernanda C. Leal, Daniela A. Rocha, Inês Lopes Cardoso

**Affiliations:** 1 FCS-UFP, Faculty of Health Sciences, Fernando Pessoa Teaching and Culture Foundation, Fernando Pessoa University, Rua Carlos da Maia 296, 4200-150 Porto, Portugal; 2 RISE-Health, Faculty of Health Sciences, Fernando Pessoa Teaching and Culture Foundation, Fernando Pessoa University, Rua Carlos da Maia 296, 4200-150 Porto, Portugal; 3 FP-I3ID, FP-BHS, Fernando Pessoa Teaching and Culture Foundation, Fernando Pessoa University, Praça de 9 de Abril 349, 4249-004 Porto, Portugal

**Keywords:** Metals, Biometals, Toxic metals, Metal chelators, Cancer, Cancer treatment

## Abstract

Biometals such as iron, copper, zinc, manganese, cobalt, and selenium play crucial roles in cellular respiration, immunity, apoptosis, and signaling. However, their excessive accumulation can induce chronic inflammation, oxidative stress, and trigger carcinogenesis. Iron addiction stands out for its importance in tumor cell replication and survival. Cancer remains one of the leading causes of mortality worldwide. Given the limitations of conventional therapies, new approaches have been explored, namely the use of metal chelators, with the aim of selectively modulating metal homeostasis in tumor cells. Metal chelation represents a promising therapeutic approach in the treatment of cancer. Metal chelators such as DFO and DFX demonstrate antineoplastic activity, although limited by low selectivity and systemic toxicity. Thiosemicarbazones, particularly Triapine, have demonstrated anticancer and antimetastatic activity in preclinical models, with clinical trials currently underway to treat tumors. SK4 appears to be a promising alternative, with greater selectivity for tumor cells, inducing apoptosis, demonstrating superior efficacy and lower toxicity. Its efficacy in preclinical models and selective profile support the need for clinical validation, potentially integrating more targeted, safe, and effective oncological therapy. Thus, the aim of this manuscript is to analyze the role of metals in cancer and evaluate the therapeutic potential of metal chelators.

## Introduction

Metals have extensive applications in industry, medicine, and agriculture. Exposure to metals can occur through several pathways, with emphasis on environmental and occupational exposure, which, when prolonged or unregulated, can result in cellular dysfunction, including carcinogenesis (Abdelaal et al. 2022; Al Osman et al. 2019; Leal et al. 2012, 2016; Souto et al. 2025).

Essential metals (or biometals), namely iron, copper, zinc, and manganese, perform biochemical and physiological functions in living organisms. For example, iron play vital roles, including its involvement in DNA synthesis resulting from the activation of the ribonucleotide reductase (RNR) enzyme, responsible to produce deoxyribonucleotides, in energy metabolism interfering with the functioning of the mitochondrial respiratory chain and in antioxidant defense. Insufficient intake of essential metals can cause disease or syndromes, but excessive intake can cause cellular damage. On the other hand, non-essential metals (or toxic metals), namely mercury, lead, cadmium, chromium, and aluminum perform no known biological functions and are known for their toxicity, even when present in extremely low concentrations. Chronic exposure to non-essential metals has been associated with the development of tumors. These metals can interfere with the homeostasis of essential metals, replacing them in enzyme catalytic centers, leading to metabolic and genotoxic dysfunctions. They can also inhibit antioxidant enzyme activities, such as catalase and superoxide dismutase (SOD), resulting in increased oxidative stress and persistent cellular damage, promoting carcinogenesis (Abdelaal et al. 2022; Al Osman et al. 2019; Balali-Mood et al. 2021; Elia et al. 2016; Kontoghiorghes 2022; Leal et al. 2020, 2023a; Pal and Firdous 2024).

Cancer represents a significant challenge to public health worldwide, affecting millions of people annually. Tumor cells exhibit iron addiction, using this metal to sustain their high rate of proliferation and energy metabolism. This dependence is evidenced by the overexpression of proteins related to iron metabolism, which facilitates the intracellular accumulation of the metal and promotes tumor progression (Abdelaal et al. 2022). In this context, metal chelators emerge as a promising strategy. These compounds can sequester metal ions essential for tumor cell proliferation, compromising their growth and metastatic capacity. Furthermore, modulation of iron and copper homeostasis can inhibit enzymes essential for cell growth, such as RNR, and reduce the production of free radicals that damage DNA (Coradduzza et al. 2024; Leal et al. 2023b, 2025; Yang et al. 2024).

This review aims to understand the influence of metals on tumor development and explore the use of metal chelators as primary or complementary therapies. Therefore, it is important to evaluate the molecular mechanisms of metal chelators, available preclinical results, safety, efficacy, and impact on tumor response. For that, the search was conducted in PubMed, ScienceDirect, Web of Science and Google Scholar databases, covering the last decade, using the keywords "metals", “biometals”, “toxic metals”, "metal chelators", “cancer”, and "cancer treatment".

## Role of metals in the main molecular pathways associated with oncogenesis

Normally, cells can repair changes that occur in DNA. However, if this damage is not repaired, it will become a mutation. If cells carrying these mutations escape cell cycle checkpoints and are not sent into apoptosis, they continue to divide, and their mutations are passed on to subsequent generations of cells. These isolated mutations, or the accumulation of mutations, lead to the development of cancer cells. Cancer is a pathology defined by uncontrolled cell proliferation that affects the normal mechanisms of cell cycle regulation, apoptosis, and differentiation. These alterations allow cancer cells to proliferate incessantly, avoid programmed cell death, and acquire invasive and metastatic capabilities. Tumor variety is a hallmark of cancer, reflecting the genetic and phenotypic variability of cells within a single tumor. This diversity contributes to heterogeneity in treatment response and disease progression (Abdelaal et al. 2022).

Cancer development can occur due to sporadic genetic changes, inherited genetic alterations (hereditary component) or epigenetic variables, such as excess weight, alcohol consumption, exposure to UV radiation, and infections. Epigenetic variations refer to alterations in DNA that do not involve changes in its sequence but affect the activity of one or more genes. These changes can influence how genes are turned on or off without altering the underlying genetic information. Epigenetics plays a crucial role in normal development, cellular differentiation, and response to environmental factors, enabling specialized cells to develop from stem cells. The main mechanisms of epigenetic variations involve DNA methylation, histone modification, chromatin remodeling, RNA regulation, genomic imprinting, and post-translational modifications of regulatory proteins (Bird 2007; Jones and Baylin 2007). Metals can modify epigenetic processes, such as DNA methylation patterns, microRNA expression, and chromatin remodulation. Such alterations contribute to the dysregulation of oncogenes and the repression of genes responsible for tumor suppression, favoring tumor initiation and progression (Balali-Mood et al. 2021; Manic et al. 2022).

Oncogenesis is driven by alterations in several molecular pathways, which confer significant advantages to cancer cells, such as uncontrolled growth, resistance to cell death, and the ability to evade the immune system. These pathways are often associated with iron metabolism and oxidative stress, processes essential for cancer cell survival and proliferation (Piccolo et al. 2024). In tumor cells, an increase in iron uptake and a decrease in its export are observed, leading to intracellular accumulation. This imbalance allows cell proliferation and DNA replication, promoting resistance to oxidative stress. Approximately 2% of human proteins depend on iron, and both iron deficiency and excess can compromise essential metabolic pathways. Tumor cells develop mechanisms to optimize iron uptake, such as overexpression of transferrin receptor 1 (TfR1) and reduction of ferroportin, the protein responsible for iron export. This leads to intracellular accumulation of this metal, favoring the synthesis of DNA and ATP, molecules essential for the accelerated cell division observed in tumors (Abdelaal et al. 2022).

### p53 pathway and its regulation by iron

Recognized as one of the key regulators of DNA damage response, the p53 tumor suppressor gene plays a central role in maintaining genomic integrity. When functional, p53 induces cell cycle arrest, cellular aging, or apoptosis. However, many tumors present mutations in the p53 gene or alterations in the regulation of the protein, which compromises its antineoplastic function (Abdelaal et al. 2022; Xu et al. 2023).

Intracellular iron directly influences p53 function by increasing oxidative stress and genomic instability, indirectly contributing to its dysfunction. One of the main mechanisms occurs through the direct interaction between heme (an iron complex with protoporphyrin IX) and the central domain of the p53 protein, which prevents its DNA binding, promoting nuclear export and, consequently, protein degradation. In situations of iron overload, intracellular heme levels increase, exacerbating this inhibitory effect. This effect has been demonstrated in cellular models, where excess iron led to a decline in p53 levels and suppression of its transcriptional function (Vávra et al. 2023).

In addition to interacting with heme, iron contributes to genomic instability through the Fenton reaction. The ferrous ion (Fe^2^⁺) reacts with hydrogen peroxide (H₂O₂), producing highly reactive hydroxyl radicals (•OH) that damage DNA and trigger repair responses mediated by proteins such as ataxia-telangiectasia and related Rad3 (ATR) and ataxia-telangiectasia mutated (ATM). These proteins activate p53 by phosphorylation. However, in chronic oxidative stress scenarios, this activation may become insufficient, contributing to mutations and functional inactivation of p53 (Chen et al. 2020).

p53 also plays a role in maintaining iron homeostasis by modulating the expression of genes associated with iron uptake, storage, and metabolism. Under normal conditions, p53 negatively regulates the expression of transferrin and positively regulates the expression of genes coding for proteins such as ferredoxin reductase (FDXR), the iron-sulfur cluster assembly unit (ISCU), and frataxin, which are essential for mitochondrial iron metabolism and the biogenesis of Fe-S clusters. Frataxin is a small mitochondrial protein crucial for cellular function, especially energy metabolism and iron regulation. Its deficiency is directly linked to Friedreich’s ataxia, a progressive genetic disease that affects the nervous system and heart. Fe-S clusters are groups of iron and sulfur atoms that act as protein cofactors. They are involved in electron transfer, enzyme catalysis, nitrogen fixation, DNA repair, and gene regulation. In tumor cells, loss of this regulation leads to mitochondrial dysfunction and increased intracellular labile iron, which exacerbates oxidative stress and favors more aggressive tumor phenotypes (Petronek et al. 2019; Zhang et al. 2022).

The correlation between iron and p53 and cell death mechanisms is particularly evident in the context of ferroptosis, an iron-dependent programmed cell death distinct from classical apoptosis. Ferroptosis is characterized by the accumulation of lipid peroxides and occurs independently of traditional cell death mechanisms. p53 can inhibit ferroptosis by activating p21 and maintaining glutathione (GSH) levels or, on the other hand, can promote it by repressing *SLC7A11* gene expression, reducing cystine uptake, and limiting the action of the antioxidant enzyme glutathione peroxidase 4 (GPX4). *SLC7A11* gene encodes the amino acid transport protein, also known as xCT, a member of the Xc-system, responsible for the import of cystine in exchange for the export of glutamate, inducing ferroptosis. This dual role of p53 in ferroptosis depends heavily on the cellular metabolic context, specific tumor type, and p53 mutational status (Liu et al. 2022a).

p53 promotes Fe^2^⁺ entry by increasing *TFR1* gene expression via regulation of long non-coding RNA (lncRNA) plasmacytoma variant translocation 1 (PVT1) and enhances mitochondrial iron biogenesis by activating SLC25A28 and ferredoxin reductase (FDXR), favoring ferroptosis. p53 induces phosphate-activated glutaminase (GLS2), increasing oxidative stress, and stimulates lipid peroxidation by activating spermidine/spermine N1-acetyltransferase 1 (SAT1) and arachidonic acid 15-lipoxygenases (ALOX15). p53 also inhibits dipeptidyl peptidase 4 (DPP4)-dependent peroxidation by relocating this protein to the nucleus and reduces GSH synthesis by repressing cystathionine-β-synthase (CBS) (via ELAVL1 or directly) and inhibiting SLC7A11, decreasing GPX4 activity and lipid peroxide elimination. Conversely, p53 can promote p21 expression, increase GSH concentration and GPX4 antioxidant activity, limiting ferroptosis (Xu et al. 2023).

In general, iron interferes with p53 function through direct and indirect mechanisms. Excess iron not only degrades p53 but creates a vicious cycle: p53 degradation increases iron accumulation; excess iron leads to oxidative stress, increasing reactive oxygen species (ROS) production through the Fenton reaction; increased ROS results in DNA damage and further p53 impairment; p53 impairment results in even less control over iron. p53 dysfunction, combined with iron overload, contributes to neoplastic development and tumor progression. The use of iron chelators allows modulation of this pathway, partially restoring p53 function and inducing antitumor responses. These data support continued research into strategies based on metal homeostasis as an innovative therapeutic approach in cancer treatment (Abdelaal et al. 2022; Xu et al. 2023).

### ROS pathway and redox signaling

As mentioned previously, iron is particularly relevant due to its ability to induce damage at high concentrations. In its ferrous state (Fe^2+^), iron is able to interact with H₂O₂ through the Fenton reaction, leading to the production of ROS, in this case the hydroxyl radical (•OH), which can cause DNA damage and genomic instability. In cancer cells, redox imbalance derives from the genesis of ROS (Arfin et al. 2021; Balali-Mood et al. 2021; Hayashi et al. 2024; Zhao et al. 2023).

Furthermore, lipid peroxidation, induced by the presence of iron and ROS, compromises the integrity of cell and mitochondrial membranes. This phenomenon can cause mitochondrial instability and the release of cytochrome c into the cytosol, activating the programmed cell death cascade. Excess iron can also induce ferroptosis (Abdelaal et al. 2022).

ROS play a crucial role in tumor biology, triggering varied effects depending on their concentration. At low concentrations, ROS act as second messengers, stimulating signaling pathways that promote cell survival, such as nuclear factor kappa B (NF-κB), mitogen-activated protein kinase (MAPK), and phosphoinositide 3-kinase/protein kinase B (PKB or Akt) (PI3K/Akt). At intermediate levels, they promote drug resistance by increasing cellular efflux. High concentrations cause DNA damage, suppress repair mechanisms, and activate mitochondrial and death receptor-dependent pathways, promoting apoptosis. ROS also stimulates autophagy by inhibiting the mammalian target of rapamycin (mTOR) and inducing genes related to autophagosome formation. Meanwhile, tumor cells can adapt to the oxidative environment by regulating antioxidant enzymes and pro-survival signaling pathways. Therefore, regulating ROS homeostasis is critical for cancer cells (Ju et al. 2024; Li et al. 2023).

The ROS-mediated redox pathway stands out for its crucial role in oncogenesis, with elevated levels of ROS arising from the exacerbated metabolism of iron and oxygen, acting as secondary messengers in several signaling pathways with oncogenic potential, including PI3K/Akt, MAPK, and NF-κB, resulting in the phosphate activation of antiapoptotic proteins and favoring tumor proliferation. These pathways activate regulatory elements that promote cellular maintenance, inflammation, and angiogenesis (Li et al. 2023).

The PI3K/Akt pathway stimulates nuclear factor erythroid 2-related factor (Nrf2): when Keap1 (Kelch protein associated with ECH) undergoes oxidation, Nrf2 migrates to the nucleus, stimulating the expression of genes encoding antioxidant enzymes such as SOD, GSH and NADPH quinone oxidoreductase (NQO1), which contributes to the stabilization of the tumor redox microenvironment. Simultaneously, the NF-κB pathway is activated by ROS, sustaining chronic inflammation, the expression of matrix metalloproteinases (MMPs), vascular endothelial growth factor (VEGF), and maintenance of telomerase reverse transcriptase (TERT), facilitating tumor replication and dissemination. Furthermore, redox inhibition of phosphatases such as phosphatase and tensin homolog deleted on chromosome 10 (PTEN) and protein phosphatase 2A (PP2A) reinforces the protumor state by persistent activation of the PI3K/Akt/mTOR pathway. To ensure their survival, tumors reinforce antioxidant systems, such as increased GSH, thioredoxin (Trx), SOD1, thioredoxin reductase (TrxR), and catalase, all under the control of Nrf2. While this response protects tumor cells, it also contributes to resistance to therapies such as chemotherapy and targeted therapies (Hayashi et al. 2024; Park et al. 2023; Wang et al. 2023a).

Another fundamental characteristic of cancer is treatment resistance. Tumor cells can develop mechanisms to mitigate the action of therapeutic agents, such as chemotherapy and radiotherapy. Notable among these mechanisms is modulation of the oxidative stress response, which involves regulating the expression of enzymes with antioxidant roles and DNA repair systems. Therefore, manipulating ROS homeostasis, whether through metal chelators or metal complexes, emerges as a promising strategy to overcome therapeutic resistance by inducing lethal levels of oxidative stress in cancer cells (Balali-Mood et al. 2021).

### NF-κB pathway and resistance to apoptosis

The NF-κB pathway plays a central role in chronic inflammation and cancer cell resistance to apoptosis. Continuous activation of this pathway, mediated by ROS and other molecules, leads to the production of anti-apoptotic proteins and inflammatory substances, creating a microenvironment favorable to tumor progression and dissemination (Ebrahimi et al. 2024).

Prolonged stimulation of the NF-κB pathway favors the maintenance of cell viability by stimulating the expression of anti-apoptotic genes, such as those encoding the B-cell lymphoma protein 2 (Bcl-2), the Bcl extra-long protein (Bcl-xL), and cellular inhibitors of apoptosis proteins (cIAPs), which interfere with the intrinsic and extrinsic mechanisms of apoptosis. Additionally, NF-κB controls the synthesis of pro-inflammatory cytokines, including interleukin-6 (IL-6) and tumor necrosis factor alpha (TNF-α), which reinforce the tumor microenvironment and promote cancer progression (Cao et al. 2024; Wu et al. 2023).

NF-κB induction is also associated to resistance to cancer therapies since it promotes the regulation of genes responsible for DNA repair and counteracts treatment-induced oxidative stress, thus reducing the effectiveness of chemotherapy and radiotherapy. This process creates a vicious cycle in which ROS production activates the NF-κB pathway, promoting cell survival and perpetuating inflammation, hindering therapeutic success. Recently, specific inhibitors of the NF-κB pathway have been designed to reverse resistance to apoptosis. These drugs block the transcription of anti-apoptotic genes and tumor inflammation modulators, improving the response to conventional treatments and offering a promising strategy for controlling tumor progression and metastasis (Abdin et al. 2021; Cao et al. 2024; Ma et al. 2024; Wu et al. 2023).

### Cell cycle modulation by ribonucleotide reductase (RNR)

RNR is a fundamental enzyme for DNA formation and is crucial for cell cycle progression, especially in the S phase. RNR catalyzes the conversion of ribonucleotides to deoxyribonucleotides, providing the building blocks necessary for DNA replication. The activity of this enzyme depends on an active metal center, which typically contains iron or manganese, facilitating the formation of free radicals essential for the catalytic reaction (Ruskoski and Boal 2021).

In cancer cells, the RNR enzyme is frequently overexpressed, namely the M1 (RRM1) and M2 (RRM2) subunits of RNR, being associated with accelerated cell proliferation and resistance to therapies. Uncontrolled tumor growth requires large amounts of new DNA, resulting in increased RNR activity (Qin et al. 2023). Blocking RNR activity, as occurs with iron chelating agents, promotes replicative stress, activating cell cycle regulatory mechanisms, such as the G1/S point (checkpoint that regulates migration from the G1 phase to the S phase of the cell cycle) and the intra-S point (checkpoint activated throughout the S phase of the cell cycle), which blocks cell cycle progression, leading to programmed cell death or, in many cases, functional cellular aging (Abdelaal et al. 2022; Morales and Xue 2021).

Furthermore, RNR is the target of multiple therapeutic strategies, including direct inhibitors such as hydroxyurea and agents that alter cellular redox status, affecting iron availability and enzyme function. Combining these approaches with conventional therapies promises to improve antitumor efficacy and alleviate treatment resistance (Li et al. 2023).

## Metal chelators as a potential cancer treatment strategy

Cancer treatment has evolved significantly, incorporating both conventional and emerging strategies. Conventional approaches, such as radiotherapy and chemotherapy, are essential pillars in the treatment of various types of cancer. However, the effectiveness of these therapies is often limited by tumor cell resistance and associated adverse effects. During these treatments, the generated ROS can induce DNA damage and promote cell apoptosis. However, elevated ROS levels can also activate signaling pathways that render neoplastic cells resistant, highlighting the need for strategies that modulate oxidative stress to improve therapeutic efficacy (Balali-Mood et al. 2021).

Among chemotherapeutic agents, the most frequently used include nucleotide analogs, such as doxorubicin and cisplatin, which generate ROS, resulting in DNA damage and inducing apoptosis. Radiotherapy, in turn, uses ionizing radiation to produce free radicals that fragment tumor DNA, leading to cell death. However, persistent activation of signaling pathways such as Nrf2, NF-κB, and PI3K/Akt in resistant cells mitigates the cytotoxic effects of these treatments, promoting DNA repair, antioxidant production, and cell survival (Dai et al. 2021; Li et al. 2024).

One of the factors related to resistance to these therapies is the induction of intrinsic antioxidant mechanisms, such as overexpression of GSH and antioxidant enzymes (SOD, catalase), which neutralize the induced oxidative stress, limiting therapeutic damage. Recently, the development of adjuvant agents that modulate redox balance, such as Nrf2 inhibitors or iron chelators, has demonstrated the potential to sensitize tumor cells, increasing the efficacy of chemotherapy and radiotherapy (Zhang et al. 2024).

### Biometal chelators

The regulation of metal metabolism, especially iron, has gained prominence as a therapeutic approach in the fight against cancer. Due to their intense metabolic activity and high proliferation rate, tumor cells demonstrate a significant dependence on this metal, making them particularly sensitive to its scarcity. In this scenario, metal chelators (substances capable of binding to free metal ions and neutralizing their biological function) have been investigated as potential agents with antitumor activity. Iron deprivation, promoted by chelators, is seen as a selective therapeutic strategy, with the potential for lower toxicity compared to conventional treatments. Iron chelation directly interferes with the activity of key enzymes, such as RNR, which are essential for DNA replication. Inhibition of this enzyme can interrupt the cell cycle at critical stages and, consequently, promote apoptosis of tumor cells. This mechanism is particularly effective in tumor cells, which are highly dependent on iron to sustain their elevated metabolism (Abdelaal and Veuger 2021; Abdelaal et al. 2022).

From a functional perspective, metal chelators can be classified into two main categories: endogenous metal chelators, naturally present in the body (such as transferrin or ferritin), which regulate the transport and storage of metals, and exogenous metal chelators, which are synthetic or natural substances administered for therapeutic purposes. Among the exogenous metal chelators, agents traditionally used to treat diseases associated with iron overload stand out, such as deferoxamine (DFO), deferasirox (DFX), and deferiprone (DFP), all approved for clinical use in pathologies such as thalassemia major and hemochromatosis (Table 1). DFO was the first metal chelator to be explored for antineoplastic purposes. Despite its efficacy in sequestering iron, its low bioavailability and the need for parenteral administration limited its application in oncology. On the other hand, DFX, an oral metal chelator with a longer half-life, has shown promising results in preclinical models and early clinical studies, particularly in the treatment of leukemias and solid tumors with active iron metabolism (Abdelaal and Veuger 2021; Abdelaal et al. 2022; Balali-Mood et al. 2021).

**Table 1:** Biometal chelators with therapeutic relevance

Biometal chelator	Target metal	Mechanism of action	Clinical/Oncological applications	Observations	References
**Deferoxamine** **(DFO)**	Iron	Sequestration of free iron; Inhibition of RNR	Thalassemia major; Hemochromatosis; Experimental use in oncology	Parenteral administration; Low bioavailability	Calabrese et al. (2020); Kontoghiorghes (2022); Morales and Xue (2021); Petronek et al. (2019); Piccolo et al. (2024); Rodriguez et al. (2024)
**Deferasirox** **(DFX)**	Iron	Iron deprivation; Indirect inhibition of cell proliferation	Leukemias; Solid tumors; Experimental use	Oral use; Prolonged half-life	Calabrese et al. (2020); Kontoghiorghes (2022); Morales and Xue (2021); Petronek et al. (2019); Piccolo et al. (2024); Rodriguez et al. (2024)
**3-Aminopyridine-2-carboxaldehyde-thiosemicarbazone (Triapine)**	Iron; Zinc	Inhibition of RNR; Blocking of DNA synthesis	Solid and hematologic tumors; Clinical study	Limiting hematologic toxicity	Rodriguez et al. (2024)
**Di-2-pyridylketone-4,4-dimethyl-3-thiosemicarbazone (Dp44mT) /** **Di-2-pyridylketone-4-cyclohexyl-4-methyl-3-thiosemicarbazone (DpC)**	Iron; Copper; Zinc	Inhibition of RNR; Oxidative stress; Selective apoptosis	Resistant solid cancers; Multiple tumor lineages	Potent activity with low hematologic toxicity	Abdelaal and Veuger (2021); Abdelaal et al. (2022); Dharmasivam et al. (2022); Krchniakova et al. (2022)
**Bis-choline tetrathiomolybdate (ATN-224)**	Copper	Inhibition of SOD1 and COX; Increased ROS and peroxynitrite	Lymphomas; KRAS-mutant carcinomas; Under study	Synergistic potential with MAPK/PI3K inhibitors	Aubert et al. (2020); Babak and Ahn (2021)
**N,N,Nʹ,Nʹ-tetrakis-(2-pyridylmethyl)ethylenediamine** **(TPEN)**	Zinc	Intracellular zinc depletion; ROS induction	Leukemia (K562 lineage); Experimental model		Mendivil-Perez et al. (2021); Yu et al. (2019)
**SK4**	Iron	Apoptosis induction; Mitochondrial metabolism inhibition	Prostate, ovarian, cervical, and triple negative breast cancer (TNBC) cell lines; Preclinical study		Abdelaal et al. (2022); Abdelaal et al. (2023)

In recent years, new chelators have emerged that expand the therapeutic strategy beyond iron, encompassing other essential metals with oncological relevance. Thiosemicarbazones such as 3-aminopyridine-2-carboxaldehyde-thiosemicarbazone (Triapine or 3-AP), di-2-pyridylketone-4,4-dimethyl-3-thiosemicarbazone (Dp44mT), and di-2-pyridylketone-4-cyclohexyl-4-methyl-3-thiosemicarbazone (DpC) form stable complexes with iron, copper, and zinc. These compounds inhibit the RNR enzyme, blocking DNA synthesis, and promote oxidative stress in tumor cells, leading to their death (Table 1). Dp44mT demonstrated high in vivo antitumor efficacy, suppressing the growth of several xenografts at lower doses than those required for Triapine, while simultaneously presenting lower hematologic toxicity. Thus, Dp44mT has demonstrated superior efficacy compared to Triapine in preclinical models. The selectivity of these molecules for tumor cells, associated with their dual mechanism of action (cytotoxic and redox) gives them potential as a targeted therapeutic agent (Dharmasivam et al. 2022; Kang et al. 2021; Krchniakova et al. 2022).

DFO and Dp44mT have also demonstrated the ability to stabilize p53, increasing its levels by inhibiting its interaction with human mouse double minute homolog 2 (MDM2), promoting its transcriptional activation. Furthermore, some of these compounds have shown efficacy even in tumors with p53 gene mutations, indicating the possible induction of alternative cellular inactivation pathways (Calabrese et al. 2020; Hino et al. 2022). DFO scavenges free iron, inhibiting vital enzymes such as RNR. In contrast, tridentate molecules such as Triapine and Dp44mT form redox-active iron-chelating complexes, inducing ROS generation, such as the hydroxyl-radical •OH, through the Fenton-like reaction, leading to apoptosis in cancer cells. The pro-oxidant effects of iron-thiosemicarbazone complexes are very important to cytotoxic action. Dp44mT stands out for its low 50% inhibitory concentration (IC₅₀) in the order of nanomoles, with antitumor efficacy in xenogeneic models using doses between 0.4–0.75 mg/kg/day, significantly reducing tumor volumes and overcoming resistance, with a controlled toxicity profile (Kang et al. 2021).

Triapine has demonstrated anticancer and antimetastatic activity in preclinical models, with phase I clinical trials (ClinicalTrials.gov ID: NCT06410248; NCT04234568) currently underway to treat tumors. Recently, new approaches have been explored, such as the use of nanomaterials and targeted approaches, such as carrier molecules and nanocarriers, that specifically target the mechanisms of iron utilization by tumor cells. Recent studies indicate that certain nanomaterials can interfere with iron homeostasis, inducing ferroptosis. Such strategies aim to increase treatment efficacy, reduce side effects, and provide more targeted and effective therapies (Li et al. 2025; Merlot et al. 2019). Controlled-release systems, such as nanomicelles combining Triapine with photoactivators, have demonstrated increased ROS formation through the Fenton reaction and induction of ferroptosis in hepatocellular carcinoma models, with more effective antitumor effects. Although still in the preclinical phase, these studies offer promising prospects for anticancer therapies (Rodriguez et al. 2024; Yu et al. 2023).

Among copper chelators, bis-choline tetrathiomolybdate (ATN-224) stands out. It inactivates crucial enzymes such as SOD1 and cytochrome c oxidase (COX), increasing the levels of reactive species such as superoxide and peroxynitrite (Table 1). These redox effects induce apoptosis in models of hematologic malignancies and lung carcinoma with Kirsten rat sarcoma viral oncogene homologue (KRAS) mutations. The metabolic dependence on copper in these cells makes them particularly sensitive to this approach, especially when combined with MAPK or PI3K pathway inhibitors, where therapeutic synergy was observed for the suppression of KRAS-mutant tumors (Aubert et al. 2020; Babak and Ahn. 2021; Nizami et al. 2023).

N,N,Nʹ,Nʹ-tetrakis-(2-pyridylmethyl)ethylenediamine (TPEN), an intracellular zinc chelator, promoted apoptosis in leukemic cells (K562) through the generation of ROS, compromising cellular redox balance (Table 1). Although less cited in recent literature, it remains relevant as a model for studying zinc-mediated redox regulation. Available data, both preclinical and early clinical, point to promising efficacy and acceptable safety profiles. However, the transition to clinical practice requires validation in controlled trials, which define doses, pharmacokinetics, and cumulative toxicity (Mendivil-Perez et al. 2021; Yu et al. 2019).

Among the new agents under investigation for metal chelation-based cancer therapy, SK4 has emerged as one of the most promising molecules (Table 1). It is a synthetic iron chelator developed to overcome the pharmacological limitations of conventional metal chelators, such as low selectivity, systemic adverse effects, and the need for prolonged administration. SK4 has a two-part structure: an iron chelation moiety and an amino acid moiety. The iron chelation part is derived from a hydroxypyridinone and is responsible for binding to ferric iron (Fe^3+^). This part is essential for the molecule’s iron-binding and cytotoxic properties. The amino acid part is a methylated analogue of L-mimosine that facilitates entry into cells through the L-type amino acid transporter 1 (LAT1). This moiety targets cells that overexpress LAT1, which is common in cancer cells. This targeting mechanism via the LAT1 transporter is the key feature that distinguishes its selectivity from traditional metal chelators like DFO and promotes preferential intracellular accumulation of SK4 in malignant tissues, increasing therapeutic efficacy and minimizing adverse effects in healthy tissues (Abdelaal et al. 2022, 2023; Kanai 2022).

Iron addiction of cancer cells represents a potential therapeutic weakness. The mechanism of action of SK4 combines multiple strands. First, its ability to sequester intracellular iron leads to the inhibition of RNR, arresting the cell cycle and inducing tumor cell apoptosis. Furthermore, iron depletion interferes with the formation of ROS, disrupting the redox balance necessary for tumor proliferation. Moreover, SK4 inhibits pro-survival signaling pathways, such as PI3K/Akt (Abdelaal et al. 2022, 2023; Morales and Xue 2021; Wang et al. 2023b).

SK4 demonstrated selective efficacy against several tumor cell lines, including breast, colon, and prostate cancers, with minimal impact on healthy cells, suggesting a favorable therapeutic index. Also, different tumor cell lines exhibit varying sensitivity to SK4, suggesting that the therapeutic efficacy may depend on the specific characteristics of each cancer type. This selectivity represents an important advantage over other antineoplastic agents, whose clinical use is often limited by systemic toxicity, and appears to be associated with the increased iron dependence observed in these cells, known as iron addiction, a characteristic that makes tumors particularly vulnerable to iron depletion (Abdelaal et al. 2022, 2023; Wang et al. 2023b).

There is evidence that SK4 may act synergistically with other therapies, such as conventional chemotherapy, enhancing its efficacy. The compound has demonstrated the ability to overcome resistance mechanisms, making it a particularly interesting candidate for the treatment of tumors refractory to standard therapies. Since the current data are primarily preclinical, continued studies in animal models and, in the future in clinical trials will be essential to confirm its efficacy, safety, and role in the therapeutic arsenal against cancer (Abdelaal et al. 2022, 2023).

Recent research emphasizes metal complexes containing ferrocenes and copper, as they can trigger ferroptosis. These complexes work by either inhibiting antioxidant enzymes (such as SOD1, TrxR, and catalase), disrupting mitochondria, or increasing ROS to a toxic level, which leads to lipid peroxidation and the depletion of molecules like NADPH and GSH, ultimately causing the death of cancer cells, including those resistant to other treatments (Guerreiro et al. 2020; Yu et al. 2023).

These advances demonstrate that pharmacological metal chelation surpasses simple iron deprivation. Agents targeting copper, zinc, and antioxidant enzymes not only modulate redox balance but also act as selective enzyme inhibitors in tumor cells. Preclinical evidence indicates that copper manipulation interferes with redox and apoptotic pathways (Abdelaal and Veuger 2021; Hu et al. 2022; Kostova 2024; Liu et al. 2022b; Mendivil-Perez et al. 2021; Paprocka et al. 2022).

### Toxic metal chelating agents

Some toxic metals, such as mercury, cadmium, and lead, accumulate in tissues after environmental or occupational exposure and can be targeted by chelating agents. In these cases, removing these metals not only reduces their genotoxic and epigenetic effects but also improves the immune response and regenerative capacity of the affected tissue, which is particularly relevant in oncological settings associated with chronic exposure to metals (Balali-Mood et al. 2021).

The main agents used in lead chelation are dimercaptosuccinic acid (DMSA, Succimer), which is effective orally for lead poisoning, and ethylenediaminetetraacetic acid (EDTA), administered intravenously in more severe cases (Table 2). Succimer has shown effectiveness in treating lead poisoning and is widely used in clinical settings. Its action is based on the formation of water-soluble complexes with the metal, facilitating renal excretion. Recent studies suggest that its combination with antioxidants may enhance the reduction of lead-induced oxidative stress, suggesting a complementary therapeutic approach with additional metabolic benefits (Balali-Mood et al. 2021, 2025; Pal and Firdous 2024; Peana et al. 2021).

**Table 2:** Toxic metal chelating agents with therapeutic relevance

Toxic metal chelating agent	Target metal	Mechanism of action	Clinical/Oncological applications	Observations	References
Dimercaptosuccinic acid (DMSA, Succimer)	Lead; Mercury	Formation of water-soluble complexes; Renal excretion	Lead and mercury poisoning; Established clinical use	Oral use; Often associated with antioxidants	Balali-Mood et al. (2025); Peana et al. (2021)
2,3-Dimercapto-1-propanesulfonic acid (DMPS)	Mercury; Lead	Complexation and excretion of toxic metals	Mercury poisoning (acute); Less commonly used in lead	High aqueous solubility; Oral and parenteral use	Balali-Mood et al. (2025); Kurt et al. (2025)
Ethylenediaminetetraacetic acid (EDTA)	Lead; Cadmium	Ionic complexation with toxic metals	Severe lead poisoning; Experimental use in cadmium	Intravenous administration; Renal effects	Manic et al. (2022); Peana et al. (2021)
D-Penicillamine (DPA)	Cadmium; Copper	Thiolated chelator; Promotes urinary excretion	Wilson’s disease; Cadmium poisoning (Experimental)	Allergenic potential; Limited use	Coradduzza et al. (2024) Pal and Firdous (2024)

For mercury, 2,3-dimercapto-1-propanesulfonic acid (DMPS) has demonstrated efficacy in both inorganic and organic forms, being used preferentially in acute poisoning (Table 2). DMSA can also be applied. DMPS, structurally similar to DMSA, but with greater aqueous solubility, can be administered orally or parenterally and has a good tolerance profile. However, despite preclinical data and favorable clinical cases, its efficacy continues to lack validation through controlled and methodologically robust clinical trials, which allow for the precise establishment of dosage, pharmacokinetics and long-term safety profile. For cadmium, despite the lack of an ideal chelator, EDTA and D-penicillamine (DPA) have demonstrated some efficacy in experimental settings (Table 2). In certain clinical situations, the combination with antioxidants has shown potential in mitigating the oxidative effects induced by these metals. Combining chelation therapy with antioxidant support may offer a dual benefit. Beyond facilitating the removal of toxic metals, this approach may help restore the activity of endogenous tumor-suppressive enzyme systems that were previously inhibited by metal binding or oxidative disruption. By alleviating this interference, normal cellular defense mechanisms can be partially reactivated, contributing to improved redox balance and genomic stability (Balali-Mood et al. 2021, 2025; Coradduzza et al. 2024; Kurt et al. 2025; Manic et al. 2022; Pal and Firdous 2024; Peana et al. 2021).

Although initial results are encouraging, the clinical use of metal chelators in oncology is still in the experimental phase for most indications. The need for more robust clinical trials, optimization of pharmacokinetic properties, and evaluation of the long-term safety profile are critical aspects for their definitive integration into clinical practice (Kontoghiorghes 2022).

## Conclusion

The role of metals in cancer development and progression has become increasingly recognized, not only due to their involvement in mechanisms such as oxidative stress, lipid peroxidation, and genomic instability, but also due to the profound changes they promote in tumor cell metabolism. Iron plays a central role in tumor biology, functioning as an essential cofactor in pathways of replication, signaling, and cell survival. Iron addiction simultaneously constitutes a vulnerability that can be therapeutically exploited through metal chelation.

Metal chelators, initially used to treat pathologies associated with metal overload, have demonstrated potential as antineoplastic agents by interfering with cellular processes essential for tumor survival. Among the best-known agents, such as DFO or DFX, significant clinical limitations have been observed, namely low selectivity, systemic toxicity, and unfavorable pharmacokinetic properties. In this context, SK4 emerges as a new-generation compound designed to overcome these obstacles, offering greater selectivity for tumor cells, action on multiple signaling pathways, and the ability to more effectively induce apoptosis. Although current data on SK4 are still preclinical, the results obtained reveal a promising profile, with high selective cytotoxic activity and potential synergism with conventional therapies. This approach represents a significant advance in oncological therapy based on the modulation of iron metabolism, paving the way for the development of more targeted strategies with lower toxicity and greater efficacy.

Thus, the use of metal chelators, with particular emphasis on innovative compounds such as SK4, may become a relevant therapeutic tool in the treatment of various types of tumors. Validation of these compounds in clinical trials will be crucial for their future integration into the oncological arsenal, potentially contributing to more personalized, effective, and safe medicine.

## Data Availability

No datasets were generated or analysed during the current study.

## References

[CR1] Abdelaal G, Veuger S (2021) Reversing oncogenic transformation with iron chelation. Oncotarget 12(2):106–124. 10.18632/oncotarget.27866 33520115 10.18632/oncotarget.27866 PMC7825639

[CR2] Abdelaal G, Carter A, Panayiotides M, Tetard D, Veuger S (2022) Novel iron chelator SK4 demonstrates cytotoxicity in a range of tumour derived cell lines. Front Mol Biosci 9:1005092. 10.3389/fmolb.2022.1005092 36213122 10.3389/fmolb.2022.1005092 PMC9540520

[CR3] Abdelaal G, Carter A, Cheung W, Panayiotidis M, Racey S, Tétard D, Veuger S (2023) Novel iron chelator SK4 drives cytotoxicity through inhibiting mitochondrial metabolism in ovarian and triple negative breast cancer cell lines. Biomedicines 11(7):2073. 10.3390/biomedicines11072073 37509712 10.3390/biomedicines11072073 PMC10377004

[CR4] Abdin SM, Tolba MF, Zaher DM, Omar HA (2021) Nuclear factor-κB signaling inhibitors revert multidrug-resistance in breast cancer cells. Chem Biol Interact 340:109450. 10.1016/j.cbi.2021.109450 33775688 10.1016/j.cbi.2021.109450

[CR5] Al Osman M, Yang F, Massey IY (2019) Exposure routes and health effects of heavy metals on children. Biometals 32(4):563–573. 10.1007/s10534-019-00193-5 30941546 10.1007/s10534-019-00193-5

[CR6] Arfin S, Jha NK, Jha SK, Kesari KK, Ruokolainen J, Roychoudhury S, Rathi B, Kumar D (2021) Oxidative stress in cancer cell metabolism. Antioxidants (Basel) 10(5):642. 10.3390/antiox10050642 33922139 10.3390/antiox10050642 PMC8143540

[CR7] Aubert L, Nandagopal N, Roux PP (2020) Targeting copper metabolism to defeat KRAS-driven colorectal cancer. Mol Cell Oncol 7(6):1822123. 10.1080/23723556.2020.1822123 33235918 10.1080/23723556.2020.1822123 PMC7671051

[CR8] Babak MV, Ahn D (2021) Modulation of intracellular copper levels as the mechanism of action of anticancer copper complexes: clinical relevance. Biomedicines 9(8):852. 10.3390/biomedicines9080852 34440056 10.3390/biomedicines9080852 PMC8389626

[CR9] Balali-Mood M, Naseri K, Tahergorabi Z, Khazdair MR, Sadeghi M (2021) Toxic mechanisms of five heavy metals: mercury, lead, chromium, cadmium, and arsenic. Front Pharmacol 12:643972. 10.3389/fphar.2021.643972 33927623 10.3389/fphar.2021.643972 PMC8078867

[CR10] Balali-Mood M, Eizadi-Mood N, Hassanian-Moghaddam H, Etemad L, Moshiri M, Vahabzadeh M, Sadeghi M (2025) Recent advances in the clinical management of intoxication by five heavy metals: mercury, lead, chromium, cadmium and arsenic. Heliyon 11(4):e42696. 10.1016/j.heliyon.2025.e42696 40040983 10.1016/j.heliyon.2025.e42696 PMC11876891

[CR11] Bird A (2007) Perceptions of epigenetics. Nature 447(7143):396–398. 10.1038/nature05913 17522671 10.1038/nature05913

[CR12] Calabrese C, Panuzzo C, Stanga S, Andreani G, Ravera S, Maglione A, Pironi L, Petiti J, Ali MSS, Scaravaglio P, Napoli F, Fava C, De Gobbi MD, Frassoni F, Saglio G, Bracco E, Pergolizzi B, Cilloni D (2020) Deferasirox-dependent iron chelation enhances mitochondrial dysfunction and restores p53 signaling by stabilization of p53 family members in leukemic cells. Int J Mol Sci 21(20):7674. 10.3390/ijms21207674 33081324 10.3390/ijms21207674 PMC7589297

[CR13] Cao Y, Yi Y, Han C, Shi B (2024) NF-κB signaling pathway in tumor microenvironment. Front Immunol 15:1476030. 10.3389/fimmu.2024.1476030 39493763 10.3389/fimmu.2024.1476030 PMC11530992

[CR14] Chen P, Tseng WH, Chi J (2020) The intersection of DNA damage response and ferroptosis-A rationale for combination therapeutics. Biology 9(8):187. 10.3390/biology9080187 32718025 10.3390/biology9080187 PMC7464484

[CR15] Coradduzza D, Congiargiu A, Azara E, Mammani IMA, De Miglio MR, Zinellu A, Carru C, Medici S (2024) Heavy metals in biological samples of cancer patients: a systematic literature review. Biometals 37(4):803–817. 10.1007/s10534-024-00583-4 38347295 10.1007/s10534-024-00583-4 PMC11254964

[CR16] Dai H, Wei Y, Liu Y, Liu J, Yu R, Zhang J, Pang J, Shao Y, Li Q, Yang Z (2021) Pathway-based analysis revealed the role of Keap1-Nrf2 pathway and PI3K-Akt pathway in Chinese esophageal squamous cell carcinoma patients with definitive chemoradiotherapy. Front Genet 12:799663. 10.3389/fgene.2021.799663 35548450 10.3389/fgene.2021.799663 PMC9081370

[CR17] Dharmasivam M, Azad MG, Afroz R, Richardson V, Jansson PJ, Richardson DR (2022) The thiosemicarbazone, DpC, broadly synergizes with multiple anti-cancer therapeutics and demonstrates temperature- and energy-dependent uptake by tumor cells. Biochim Biophys Acta Gen Sub 1866(8):130152. 10.1016/j.bbagen.2022.130152 10.1016/j.bbagen.2022.130152 35436509

[CR18] Ebrahimi N, Abdulwahid ARR, Mansouri A, Karimi N, Bostani RJ, Beiranvand S, Adelian S, Khorram R, Vafadar R, Hamblin MR, Aref AR (2024) Targeting the NF-κB pathway as a potential regulator of immune checkpoints in cancer immunotherapy. Cell Mol Life Sci 81(1):106. 10.1007/s00018-023-05098-8 38418707 10.1007/s00018-023-05098-8 PMC10902086

[CR19] Elia I, Schmieder R, Christen S, Fendt S (2016) Organ-specific cancer metabolism and its potential for therapy. Handb Exp Pharmacol 233:321–353. 10.1007/164_2015_10 25912014 10.1007/164_2015_10

[CR20] Guerreiro JF, Gomes MAGB, Pagliari F, Jansen J, Marafioti MG, Nistico C, Hanley R, Costa RO, Ferreira SS, Mendes F, Fernandes C, Horn A, Tirinato L, Seco J (2020) Iron and copper complexes with antioxidant activity as inhibitors of the metastatic potential of glioma cells. RSC Adv 10(22):12699–12710. 10.1039/d0ra00166j 35492123 10.1039/d0ra00166j PMC9051468

[CR21] Hayashi M, Okazaki K, Papgiannakopoulos T, Motohashi H (2024) The complex roles of redox and antioxidant biology in cancer. Cold Spring Harb Perspect Med 14(11):a041546. 10.1101/cshperspect.a041546 38772703 10.1101/cshperspect.a041546 PMC11529857

[CR22] Hino K, Yanatori I, Hara Y, Nishina S (2022) Iron and liver cancer: an inseparable connection. FEBS J 289(24):7810–7829. 10.1111/febs.16208 34543507 10.1111/febs.16208

[CR23] Hu H, Xu Q, Mo Z, Hu X, He Q, Zhang Z, Xu Z (2022) New anti-cancer explorations based on metal ions. J Nanobiotechnol 20(1):457. 10.1186/s12951-022-01661-w 10.1186/s12951-022-01661-w PMC9590139 36274142

[CR24] Jones PA, Baylin SB (2007) The epigenomics of cancer. Cell 128(4):683–692. 10.1016/j.cell.2007.01.029 17320506 10.1016/j.cell.2007.01.029 PMC3894624

[CR25] Ju S, Singh MK, Han S, Ranbhise J, Ha J, Choe W, Yoon K, Yeo SG, Kim SS, Kang I (2024) Oxidative stress and cancer therapy: controlling cancer cells using reactive oxygen species. Int J Mol Sci 25(22):12387. 10.3390/ijms252212387 39596452 10.3390/ijms252212387 PMC11595237

[CR26] Kanai Y (2022) Amino acid transporter LAT1 (SLC7A5) as a molecular target for cancer diagnosis and therapeutics. Pharmacol Ther 230:107964. 10.1016/j.pharmthera.2021.107964 34390745 10.1016/j.pharmthera.2021.107964

[CR27] Kang YJ, Holley CK, Abidian MR, Madhankumar AB, Connor J, Majd S (2021) Tumor targeted delivery of an anti-cancer therapeutic: an in vitro and in vivo evaluation. Adv Healthc Mater 10(2):e2001261. 10.1002/adhm.202001261 33191612 10.1002/adhm.202001261

[CR28] Kontoghiorghes GJ (2022) New iron metabolic pathways and chelation targeting strategies affecting the treatment of all types and stages of cancer. Int J Mol Sci 23(22):13990. 10.3390/ijms232213990 36430469 10.3390/ijms232213990 PMC9696688

[CR29] Kostova I (2024) Editorial: Metallodrugs in cancer therapy: past, present and new strategies. Front Chem 12:1428502. 10.3389/fchem.2024.1428502 38882213 10.3389/fchem.2024.1428502 PMC11177608

[CR30] Krchniakova M, Paukovcekova S, Chlapek P, Neradil J, Skoda J, Veselska R (2022) Thiosemicarbazones and selected tyrosine kinase inhibitors synergize in pediatric solid tumors: NDRG1 upregulation and impaired prosurvival signaling in neuroblastoma cells. Front Pharmacol 13:976955. 10.3389/fphar.2022.976955 36160437 10.3389/fphar.2022.976955 PMC9490180

[CR31] Kurt F, Akcil A, Cangur S, Yıldız M (2025) Evaluation of mass mercury poisoning cases occurring in a center in Turkiye: symptomatology, treatment methods, and follow-up processes. Eur J Pediatr 184(5):309. 10.1007/s00431-025-06143-3 40266418 10.1007/s00431-025-06143-3 PMC12018509

[CR32] Leal MFC, Catarino MIL, Pimenta AM, Souto MRS, Pinheiro TSN (2012) Speciation of copper and zinc in urine–importance of metals in neurodegenerative diseases. Quim Nova 35(10):1985–1990. 10.1590/S0100-40422012001000018

[CR33] Leal MFC, Catarino RIL, Pimenta AM, Souto MRS, Afonso CS, Fernandes AFQ (2016) Lead migration from toys by anodic stripping voltammetry using a bismuth film electrode. Arch Environ Occup Health 71(5):300–306. 10.1080/19338244.2015.1096757 26402643 10.1080/19338244.2015.1096757

[CR34] Leal MFC, Catarino RIL, Pimenta AM, Souto MRS (2020) Roles of metal microelements in neurodegenerative diseases. Neurophysiology 52(1):80–88. 10.1007/s11062-020-09854-5

[CR35] Leal MFC, Catarino RIL, Pimenta AM, Souto MRS (2023a) The influence of the biometals Cu Fe, and Zn and the toxic metals Cd and Pb on human health and disease. Trace Elem Electrol 40(1):1–22. 10.5414/TE500038

[CR36] Leal MFC, Catarino RIL, Pimenta AM, Souto MRS (2023b) Metal chelators as part of a strategy for the treatment of neurodegenerative diseases. Trace Elem Electrol 40(3):126–138. 10.5414/TE500056

[CR37] Leal MFC, Duarte RJG, Lopes Cardoso I, Catarino RIL, Pimenta AM, Souto MRS (2025) Bioactive compounds from marine macroalgae in the treatment and prevention of neurodegenerative diseases. Neurophysiology. 10.1007/s11062-025-09953-1

[CR38] Li Y, Zhang X, Wang Z, Li B, Zhu H (2023) Modulation of redox homeostasis: a strategy to overcome cancer drug resistance. Front Pharmacol 14:1156538. 10.3389/fphar.2023.1156538 37033606 10.3389/fphar.2023.1156538 PMC10073466

[CR39] Li Y, Zhao B, Peng J, Tang H, Wang S, Peng S, Ye F, Wang J, Ouyang K, Li J, Cai M, Chen Y (2024) Inhibition of NF-κB signaling unveils novel strategies to overcome drug resistance in cancers. Drug Resist Updat 73:101042. 10.1016/j.drup.2023.101042 38219532 10.1016/j.drup.2023.101042

[CR40] Li B, Zhang B, Cheng Z, Lou Y, Chen S (2025) Nanomaterials targeting iron homeostasis: a promising strategy for cancer treatment. Front Bioeng Biotechnol 13:1511197. 10.3389/fbioe.2025.1511197 40144390 10.3389/fbioe.2025.1511197 PMC11937013

[CR41] Liu M, Kong X, Yao Y, Wang X, Yang W, Wu H, Li S, Ding J, Yang J (2022a) The critical role and molecular mechanisms of ferroptosis in antioxidant systems: a narrative review. Ann Transl Med 10(6):368. 10.21037/atm-21-6942 35434035 10.21037/atm-21-6942 PMC9011221

[CR42] Liu W, Zhao Y, Wang G, Feng S, Ge X, Ye W, Wang Z, Zhu Y, Cai W, Bai J, Zhou X (2022b) TRIM22 inhibits osteosarcoma progression through destabilizing NRF2 and thus activation of ROS/AMPK/mTOR/autophagy signaling. Redox Biol 53:102344. 10.1016/j.redox.2022.102344 35636015 10.1016/j.redox.2022.102344 PMC9144049

[CR43] Ma Q, Hao S, Hong W, Tergaonkar V, Sethi G, Tian Y, Duan C (2024) Versatile function of NF-ĸB in inflammation and cancer. Exp Hematol Oncol 13(1):68. 10.1186/s40164-024-00529-z 39014491 10.1186/s40164-024-00529-z PMC11251119

[CR44] Manić L, Wallace D, Onganer PU, Taalab YM, Farooqi AA, Antonijević B, Djordjevic AB (2022) Epigenetic mechanisms in metal carcinogenesis. Toxicol Rep 9:778–787. 10.1016/j.toxrep.2022.03.037 36561948 10.1016/j.toxrep.2022.03.037 PMC9764177

[CR45] Mendivil-Perez M, Velez-Pardo C, David-Yepes GE, Fox JE, Jimenez-Del-Rio M (2021) TPEN exerts selective anti-leukemic efficacy in ex vivo drug-resistant childhood acute leukemia. Biometals 34(1):49–66. 10.1007/s10534-020-00262-0 33098492 10.1007/s10534-020-00262-0

[CR46] Merlot AM, Kalinowski DS, Kovacevic Z, Jansson PJ, Sahni S, Huang ML, Lane DJR, Lok H, Richardson DR (2019) Exploiting cancer metal metabolism using anti-cancer metal-binding agents. Curr Med Chem 26(2):302–322. 10.2174/0929867324666170705120809 28685681 10.2174/0929867324666170705120809

[CR47] Morales M, Xue X (2021) Targeting iron metabolism in cancer therapy. Theranostics 11(17):8412–8429. 10.7150/thno.59092 34373750 10.7150/thno.59092 PMC8344014

[CR48] National Cancer Institute (NCI). Testing the addition of an anti-cancer drug, triapine, to the usual radiation-based treatment (Lutetium Lu 177 Dotatate) for neuroendocrine tumors. ClinicalTrials.gov; 2025. ID: NCT04234568.

[CR49] Nizami ZN, Aburawi HE, Semlali A, Muhammad K, Iratni R (2023) Oxidative stress inducers in cancer therapy: preclinical and clinical evidence. Antioxidants (Basel) 12(6):1159. 10.3390/antiox12061159 37371889 10.3390/antiox12061159 PMC10295724

[CR50] Northwestern University. Triapine in combination with temozolomide for the treatment of patients with recurrent Glioblastoma. ClinicalTrials.gov; 2025. ID: NCT06410248.

[CR51] Pal S, Firdous SM (2024) Unraveling the role of heavy metals xenobiotics in cancer: a critical review. Discov Oncol 15(1):615. 10.1007/s12672-024-01417-y 39495398 10.1007/s12672-024-01417-y PMC11535144

[CR52] Paprocka R, Wiese-Szadkowska M, Janciauskiene S, Kosmalski T, Kulik M, Helmin-Basa A (2022) Latest developments in metal complexes as anticancer agents. Coord Chem Rev 452:214307. 10.1016/j.ccr.2021.214307

[CR53] Park SY, Gurung R, Hwang JH, Kang J, Jung HJ, Zeb A, Hwang J, Park SJ, Maeng H, Shin D, Oh SH (2023) Development of KEAP1-targeting PROTAC and its antioxidant properties: In vitro and in vivo. Redox Biol 64:102783. 10.1016/j.redox.2023.102783 37348157 10.1016/j.redox.2023.102783 PMC10333676

[CR54] Peana M, Pelucelli A, Medici S, Cappai R, Nurchi VM, Zoroddu MA (2021) Metal toxicity and speciation: a review. Curr Med Chem 28(35):7190–7208. 10.2174/0929867328666210324161205 33761850 10.2174/0929867328666210324161205

[CR55] Petronek MS, Spitz DR, Buettner GR, Allen BG (2019) Linking cancer metabolic dysfunction and genetic instability through the lens of iron metabolism. Cancers (Basel) 11(8):1077. 10.3390/cancers11081077 31366108 10.3390/cancers11081077 PMC6721799

[CR56] Piccolo M, Ferraro MG, Iazzetti F, Santamaria R, Irace C (2024) Insight into iron, oxidative stress and ferroptosis: therapy targets for approaching anticancer strategies. Cancers (Basel) 16(6):1220. 10.3390/cancers16061220 38539554 10.3390/cancers16061220 PMC10969343

[CR57] Qin Z, Xie B, Qian J, Ma X, Zhang L, Wei J, Wang Z, Fan L, Zhu Z, Qian Z, Yin H, Zhu F, Tan Y (2023) Over-expression of RRM2 predicts adverse prognosis correlated with immune infiltrates: a potential biomarker for hepatocellular carcinoma. Front Oncol 13:1144269. 10.3389/fonc.2023.1144269 37056349 10.3389/fonc.2023.1144269 PMC10086364

[CR58] Rodríguez I, Acosta C, Nieves-Escobar C, Strangmark E, Claudio-Ares O, Figueroa AIV, Soto-Millán AM, Orta-Rivera AM, Astashkin AV, Tinoco AD (2024) DefNEtTrp: an iron dual chelator approach for anticancer application. JACS Au 4(12):4799–4808. 10.1021/jacsau.4c00774 39735911 10.1021/jacsau.4c00774 PMC11672142

[CR59] Ruskoski TB, Boal AK (2021) The periodic table of ribonucleotide reductases. J Biol Chem 297(4):101137. 10.1016/j.jbc.2021.101137 34461093 10.1016/j.jbc.2021.101137 PMC8463856

[CR60] Souto MRS, Pimenta AM, Catarino RI, Leal MFC, Simões ETR (2025) Heavy metals in milk and dairy products: safety and analysis. Pollutants 5:29. 10.3390/pollutants5030029

[CR61] Vávra J, Sergunin A, Pompach P, Savchenko D, Hraníček J, Šloufová I, Shimizu T, Martínková M (2023) Characterization of the interaction between the tumour suppressor p53 and heme and its role in the protein conformational dynamics studied by various spectroscopic techniques and hydrogen/deuterium exchange coupled with mass spectrometry. J Inorg Biochem 243:112180. 10.1016/j.jinorgbio.2023.112180 36934467 10.1016/j.jinorgbio.2023.112180

[CR62] Wang R, Liang L, Matsumoto M, Iwata K, Umemura A, He F (2023a) Reactive oxygen species and NRF2 signaling, friends or foes in cancer? Biomolecules 13(2):353. 10.3390/biom13020353 36830722 10.3390/biom13020353 PMC9953152

[CR63] Wang R, Li J, Fu Y, Li Y, Qi Y, Li C, Gao F, Li C (2023b) Ferritinophagy-mediated apoptosis and paraptosis induction involved MAPK and PI3K/AKT pathway in mechanism of an iron chelator. Biochem Pharmacol 218:115874. 10.1016/j.bcp.2023.115874 37866802 10.1016/j.bcp.2023.115874

[CR64] Wu X, Sun L, Xu F (2023) NF-κB in cell deaths, therapeutic resistance and nanotherapy of tumors: recent advances. Pharmaceuticals (Basel) 16(6):783. 10.3390/ph16060783 37375731 10.3390/ph16060783 PMC10302521

[CR65] Xu R, Wang W, Zhang W (2023) Ferroptosis and the bidirectional regulatory factor p53. Cell Death Discov 9(1):197. 10.1038/s41420-023-01517-8 37386007 10.1038/s41420-023-01517-8 PMC10310766

[CR66] Yang Y, Fan H, Guo Z (2024) Modulation of metal homeostasis for cancer therapy. ChemPlusChem 89(6):e202300624. 10.1002/cplu.202300624 38315756 10.1002/cplu.202300624

[CR67] Yu Z, Yu Z, Chen Z, Yang L, Ma M, Lu S, Wang C, Teng C, Nie Y (2019) Zinc chelator TPEN induces pancreatic cancer cell death through causing oxidative stress and inhibiting cell autophagy. J Cell Physiol 234(11):20648–20661. 10.1002/jcp.28670 31054150 10.1002/jcp.28670

[CR68] Yu H, Yan J, Li Z, Yang L, Ju F, Sun Y (2023) Recent trends in emerging strategies for ferroptosis-based cancer therapy. Nanoscale Adv 5(5):1271–1290. 10.1039/d2na00719c 36866253 10.1039/d2na00719c PMC9972547

[CR69] Zhang Y, Li M, Guo Y, Liu S, Tao Y (2022) The organelle-specific regulations and epigenetic regulators in Ferroptosis. Front Pharmacol 13:905501. 10.3389/fphar.2022.905501 35784729 10.3389/fphar.2022.905501 PMC9247141

[CR70] Zhang Z, Gao J, Jia L, Kong S, Zhai M, Wang S, Li W, Wang S, Su Y, Li W, Zhu C, Wang W, Lu Y, Li W (2024) Excessive glutathione intake contributes to chemotherapy resistance in breast cancer: a propensity score matching analysis. World J Surg Oncol 22(1):345. 10.1186/s12957-024-03626-9 39709466 10.1186/s12957-024-03626-9 PMC11663319

[CR71] Zhao Y, Ye X, Xiong Z, Ihsan A, Ares I, Martínez M, Lopez-Torres B, Martínez-Larrañaga M, Anadón A, Wang X, Martínez M (2023) Cancer metabolism: the role of ROS in DNA damage and induction of apoptosis in cancer cells. Metabolites 13(7):796. 10.3390/metabo13070796 37512503 10.3390/metabo13070796 PMC10383295

